# The composition of the arbuscular mycorrhizal fungal bacteriome is species dependent

**DOI:** 10.1186/s40793-024-00623-z

**Published:** 2024-10-16

**Authors:** Zakaria Lahrach, Jean Legeay, Bulbul Ahmed, Mohamed Hijri

**Affiliations:** 1 Institut de Recherche en Biologie Végétale, Département de Sciences Biologiques, Université de Montréal, 4101 East Sherbrooke St., Montréal, QC Canada; 2 African Genome Center, University Mohammed VI Polytechnic (UM6P), Ben Guerir, 43150 Morocco

**Keywords:** Arbuscular mycorrhizal fungi, Bacteriome core, Community structure, Symbiosis

## Abstract

**Background:**

In addition to their role as endosymbionts for plant roots, arbuscular mycorrhizal fungi (AMF) engage in complex interactions with various soil microorganisms, the rhizosphere, and the root endosphere of host plants. They also host diverse prokaryotic groups within their mycelia, contributing to what is termed multipartite symbiosis. In this study, we examined the impact of three AMF species—*Rhizophagus irregularis*,* R. clarus*, and *R. cerebriforme*—combined with microbial bioaugmentation on the diversity and composition of bacterial communities in the mycelia and hyphosphere. Using a microcosm design to separate the influence of host plant roots from AMF mycelia and Illumina MiSeq amplicon sequencing to analyze the bacterial communities.

**Results:**

Our results revealed that, while AMF identity and microbial bioaugmentation did not affect the structure of bacterial communities in the hyphosphere soil, they significantly altered the communities associated with their mycelia. Although all three AMF species belong to the same genus, with *R. irregularis* and *R. clarus* being closely related compared to *R. cerebriforme*, we observed variations in the bacterial communities associated with their mycelia. Interestingly, the mycelial bacterial community of *R. cerebriforme* contained 60 bacteriome core taxa exclusive to it, while *R. clarus* and *R. irregularis* had 25 and 9 exclusive taxa, respectively.

**Conclusion:**

This study suggests that organismal phylogeny influences the bacterial communities associated with AMF mycelia. These findings provide new insights into AMF and bacterial interactions, which are crucial for the successful deployment of AMF inoculants. The taxonomic diversity of AMF inoculants is important for engineering the plant microbiome and enhancing ecosystem services.

**Supplementary Information:**

The online version contains supplementary material available at 10.1186/s40793-024-00623-z.

## Background

Among the fungi that establish mycorrhizal symbiosis, arbuscular mycorrhizal fungi (AMF) are prominent obligate biotrophs that form symbiotic relationships with the roots of 72% of terrestrial plants. This mutualism is crucial for nutrient exchange, as it enhances plant access to essential minerals like phosphorus and nitrogen while providing fungi with carbon-rich compounds from photosynthesis [1]. The interactions between AMF and various plant species have been extensively documented, and these symbiotic relationships have great potential to improve the efficiency, resilience, and sustainability of agricultural ecosystems [2–4]. The ecological success of AMF in acquiring nutrients through extraradical hyphae that explore the soil, alleviating abiotic stress, mitigating pathogen attacks, and promoting hyphal growth is largely due to beneficial soil microbial communities that closely or loosely interact with AMF mycelia [5, 6]. Genome sequencing revealed that the members of the Glomeromycotina lineage are deficient in genes involved in carbon assimilation, such as lignocellulose degradation, fatty acid and secondary metabolite toxin synthesis, and thiamin biosynthesis [7–10]. AMF also lack the ability to secrete invertase or sucrose transporters and the capacity to mineralize insoluble nutrients [11]. AMF compensate for these deficiencies by developing obligate symbiosis with plants and by harboring or recruiting beneficial soil microbiota, especially bacteria.

Arbuscular mycorrhizal fungi interact with various microorganisms in the rhizosphere to form their own community of mycorrhizobiota, which includes diverse and abundant bacterial communities living in the mycorrhizosphere. The interaction of AMF with bacteria occur at varying areas in soil and roots [12]. These bacterial communities can be found in/on mycorrhizal roots, spores, sporocarps, and extraradical hyphae [13]. They include cultivable bacteria in the mycorrhizosphere and virtually uncultivable endobacteria living in spores and mycelia [14]. Several of these bacteria have been identified and characterized on the surface and inside of AMF spores and hyphae [5, 15, 16]. *Geosiphon pyriformis* is an unusual AMF species as it forms a symbiosis with cyanobacteria rather than plants. *G. pyriformis* establishes an endosymbiotic relationship with the cyanobacterium *Nostoc punctiforme*, where the cyanobacterium supplies organic compounds to the host fungus, which in turn provides minerals and water [17, 18]. Interactions between phosphate-solubilizing bacteria and AMF are important for plant phosphorus (P) acquisition, enabling efficient mobilization and transport to plant roots [19–22]. Bacterial isolates from the mycorrhizosphere and hyphosphere of AMF, including *Asia lannaensis*, *Rahnella* sp., *Pantoea* sp., *Pseudomonas* sp., and *Burkhoderia* sp., displayed phosphate rock solubilization ability [23, 24]. Therefore, the inoculation of plants with AMF and bacteria resulted in better plant performance and higher P use efficiency compared to non-inoculated plants and those inoculated with either AMF or bacteria [25]. The interplay between AMF and microbiota reduced nitrous oxide emissions after nitrogen fertilizer application [26, 27].

Conversely, the interactions of AMF with microbial communities do not always translate into gains for the fungus and host plant. AMF may serve as a pathway for the invasion of mycoparasites, opportunistic plant root colonizers, and phytopathogens. By employing a microscopic camera-imaging system coupled with succinate dehydrogenase staining, De Jaeger et al. [28] tracked the penetration of *Trichoderma harzianum* through the extra-radical mycelium of *Glomus* sp. MUCL 41,833 before invading host plant root cells. Consequently, *T. harzianum* induced protoplasm degradation and reduced the viability of extraradical and intraradical mycelia, and spores in the fungal host [28]. Furthermore, Hijri et al. [29] isolated *Leptosphaeria* sp. from the spores of AMF and found that they were pathogens of Brassicaceae, although the study was not conclusive on the pathogenicity of this fungus. An in vitro study showed that *Trichoderma atroviride* PKI1 parasitized *Gigaspora gigantea* and *G. margarita* hyphae by degrading the cell walls before causing the death of *Medicago trunculata* root cells. *Trichoderma atroviride* colonized plant roots independently of the fungal route since the AMF colonization did not affect root or hyphae colonization by *T. atrovide* [30].

Despite the plethora of evidence supporting the importance of microbes associated with the performance and fitness of mycorrhizal symbiosis [6], interactions between AMF and other microbes are often overlooked in the development and management of AMF-based inoculants. An recent analysis of commercial AMF inoculants by Basiru et al. [3] indicated that only 19% comprised microbial consortia other than the active ingredients. Supplementing selected growth-promoting bacteria to AMF inoculants can improve the establishment of AMF in plant roots. Additionally, these microbes contribute to plant growth by fixing nutrients, solubilizing and mobilizing minerals, biocontrolling fungi and plant pathogens, producing phytohormones, forming biofilms, and degrading cellulose and toxins [31]. There are still ongoing debates on whether in vitro propagated inoculants maintain higher colonization efficiency over time compared to their in vivo counterparts and whether fungal species’ diversity or composition is more important [3]. Therefore, to improve the performance of AMF inoculants, proper attention needs to be given to bacterial communities interacting with AMF when formulating AMF inoculants, particularly those produced in vitro in sterile conditions, as well as when planning field management. A consortium of bacteria and AMF interacting in natural environments is likely to perform better than combining two microbes belonging to different environmental and ecological niches. Furthermore, it is crucial to unravel the ecological factors that mediate AMF symbiosis, as they are often linked to agronomic management practices. Knowledge of the factors that determine which bacterial communities are selected by certain AMF genera under certain soil conditions will also enable the production of inoculants and design of management plans to be tailored for a more specific purpose.

Although numerous studies have investigated multipartite symbiosis involving AMF, plants, and bacteria, there has been a bias towards AMF–plant associations [32]. Consequently, there remains a limited understanding of the driving forces behind the interactions between mycorrhizal fungi and bacteria, as well as the enduring impacts of AMF or bacteria inoculation on soil microbial communities. Therefore, there is a necessity for research to delve into the mechanisms underlying AMF–bacteria interactions. Many studies have provided valuable insights into the interactions between AMF and bacteria, elucidating not only their establishment within host plants but also the mechanisms of interaction that influence other microbes residing in the rhizosphere. However, most existing studies on plant–AMF–bacterial interactions include the influence of roots, which could potentially mask the effect of AMF mycelia [32]. The aim of this study was to investigate how three AMF species—*Rhizophagus irregularis*,* R. clarus*, and *R. cerebriforme*—and microbial bioaugmentation affect the host plant and the diversity and composition of bacterial communities in both mycelia and hyphosphere soil. These species were selected because they were cultivated in vitro using a similar root organ culture, with *R. irregularis* being one of the most important species widely utilized as a commercial inoculant [3]. We hypothesized that: (i) microbial suspension amendment from natural soil will increase the bacterial diversity and alter the community structure; (ii) AMF identity will influence both the mycelia and hyphosphere bacterial community structures; and (iii) AMF species will select distinct bacterial communities. To test these hypotheses, we employed a microcosm design to isolate the effects of host plant roots from those of AMF mycelia. We conducted a greenhouse trial incorporating two factors: three distinct AMF species and two levels of microbial inoculation. Bacterial diversity and community structure were assessed using Illumina MiSeq amplicon sequencing targeting the bacterial 16 S rRNA gene. The findings of this study will offer valuable insights into how the identity of AMF influences bacterial communities, potentially revolutionizing bioinoculant engineering for agricultural and environmental applications.

## Materials and methods

### Experimental design

**Fig. 1:**
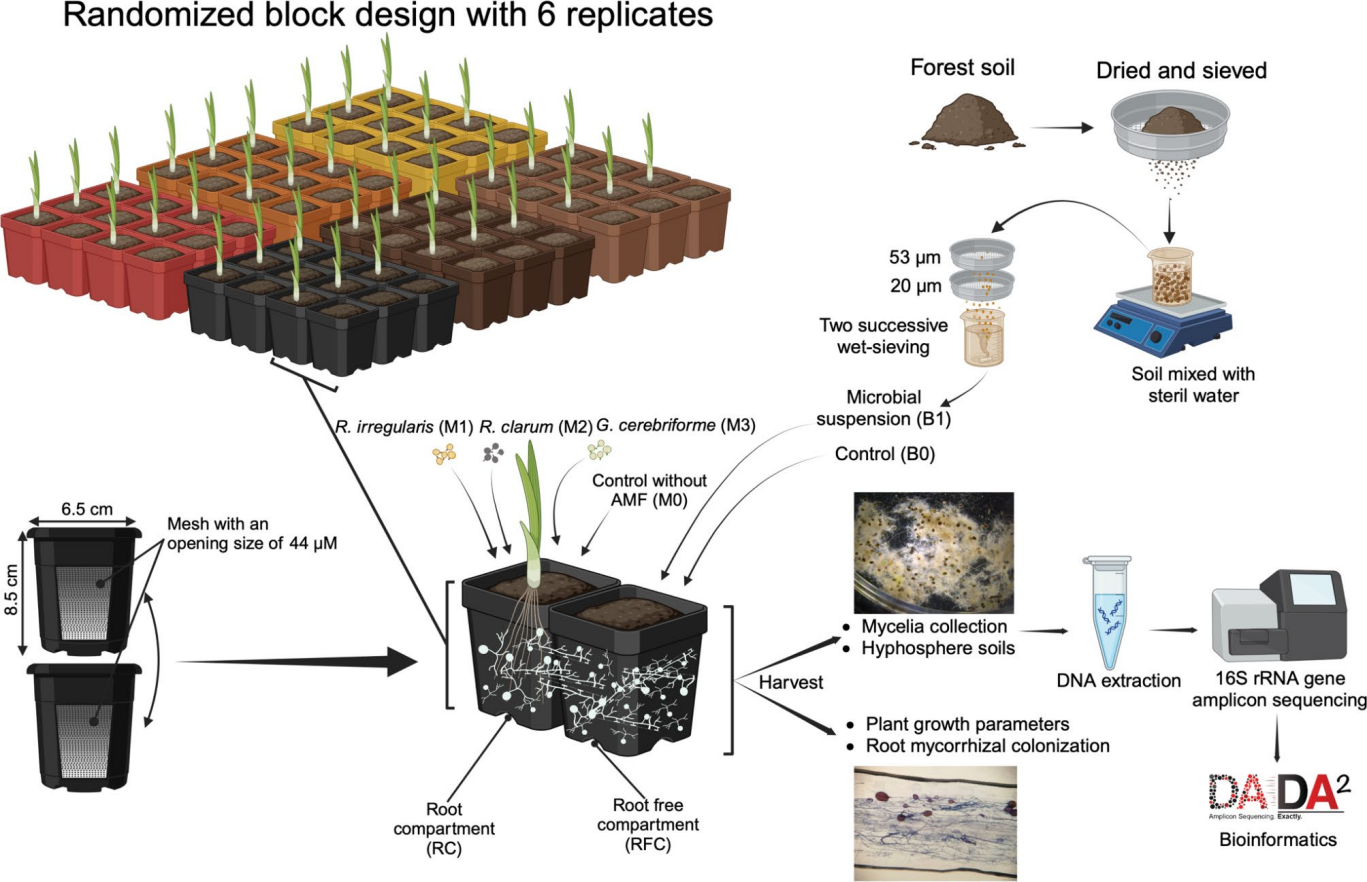
An overview of the experimental design, the arrangement of the microcosm units, and the treatments. This figure was created using Biorender

To examine the influence of AMF species and microbial inoculation with a soil suspension on the bacterial community structure associated with their mycelia, we conducted a greenhouse experiment. We employed a microcosm setup consisting of two pots (6.5 × 6.5 × 8.5 cm each), each featuring a cut side and a mesh nylon membrane of 44 μm. These pots were then attached to each other and sealed with silicone adhesive to create a unit with two compartments, allowing connectivity through two nylon membranes (Fig. 1). One pot was designated for plant growth (root compartment, RC), while the other facilitated hyphal proliferation (root-free compartment, RFC) and allowed microbial inoculation with a soil suspension. Both pots were interconnected through two 44 μm membrane layers (SEFAR Incorporation, Buffalo, NY, USA), allowing only the passage of hyphae while excluding roots.

We utilized a randomized block design with six replicates, incorporating two factors: (1) three distinct AMF species (M1: *Rhizophagus irregularis* isolate DAOM 197198, M2: *Rhizophagus clarus* isolate DAOM 234179, and M3: *Rhizophagus cerebriforme* isolate DAOM 227022, all cultivated in vitro under identical conditions) provided by Premier Tech, Rivières du Loup, QC, Canada; and (2) two levels of microbial inoculation (B0 and B1). This resulted in six treatment combinations: M1B0, M1B1, M2B0, M2B1, M3B0, and M3B1. Additionally, two control groups without AMF spore inoculation (M0) and with autoclaved distilled water (B0) were included to evaluate plant growth and root colonization, resulting in two combinations (M0B0 and M0B1). The experiment was arranged on a mesh table to prevent cross-contamination between experimental units via water.

The microbial suspension was made from soil collected from the rhizosphere of an old maple forest at the Gault Nature Reserve in Mont Saint-Hilaire, QC, Canada [33, 34]. This soil was selected because it is known for hosting a diverse AMF community, thereby increasing the likelihood of fostering highly interactive microbial communities with the AMF species utilized in this experiment. A composite sample was created by combining four soil samples obtained from the forest, which were then sieved at 2 mm to remove large particles. The soil was mixed with sterilized distilled water, agitated for 30 min to achieve suspension, and left at room temperature for at least 2 h. The suspension was subsequently filtered through 53 and 20 μm sterilized sieves and collected in a sterilized Erlenmeyer flask to remove AMF spores (Fig. 1). The microbial suspension (filtrate) was confirmed to be free from AMF spores through binocular observation on a Petri dish. The suspension was used to inoculate the pot in the root-free compartment as described below.

**Fig. 2:**
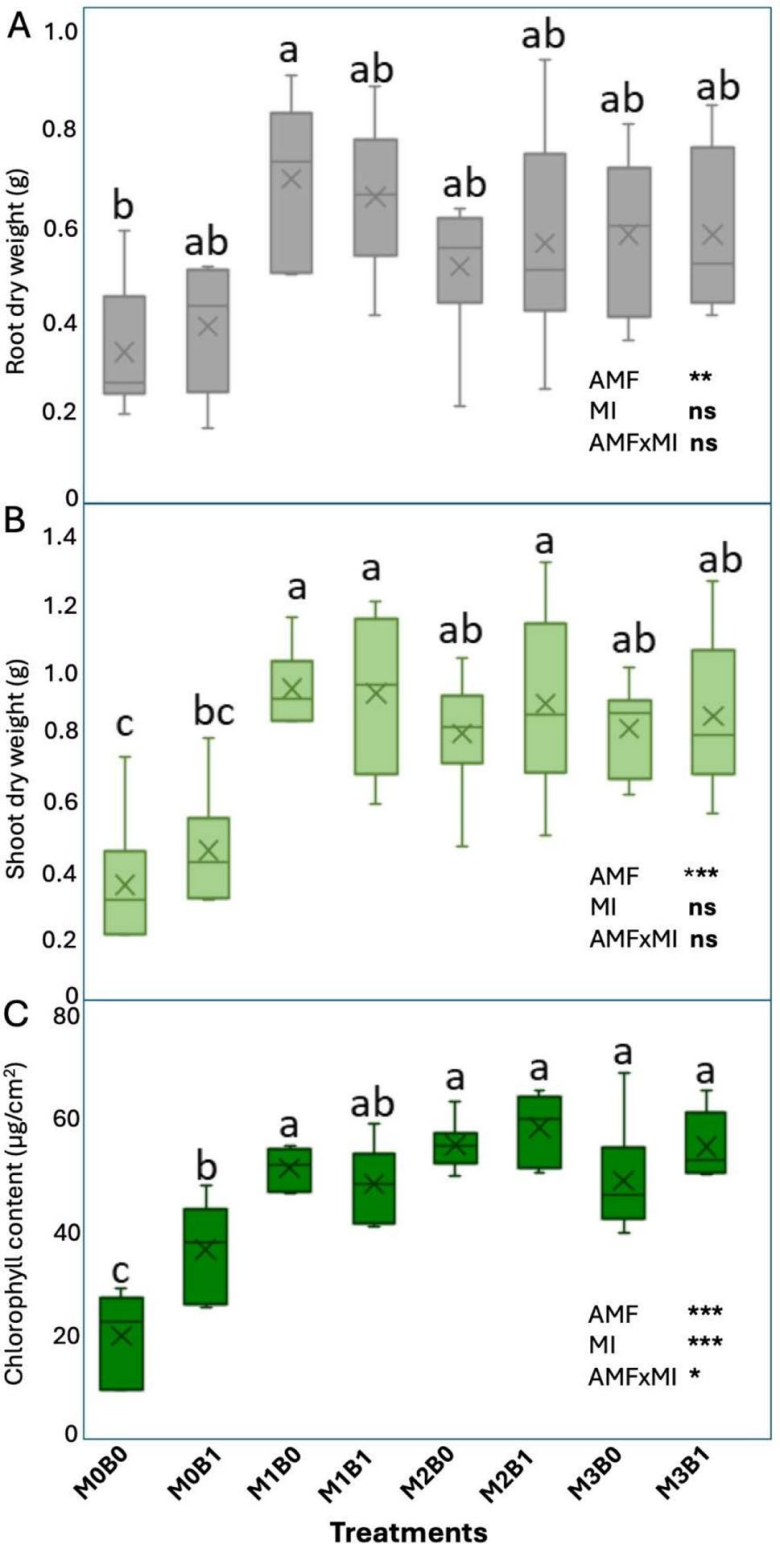
The effects of arbuscular mycorrhizal fungi (AMF) identity and microbial inoculation (MI) on various parameters related to leek plants were evaluated, including: (**A**) root dry weight, (**B**) shoot dry weight, and (**C**) chlorophyll content. The treatments included: M0B0 (control without any inoculation), M0B1 (microbial inculation without AMF), M1B0 (inoculation with *R. irregularis* without MI), M1B1 (inoculation with *R. irregularis* with MI), M2B0 (inoculation with *R. clarus* without MI), M2B1 (inoculation with *R. clarus* with MI), M3B0 (inoculation with *R. cerebriforme* without MI), and M3B1 (inoculation with *R. cerebriforme* with MI). The significance levels are denoted as follows: * *p* < 0.05, ** *p* < 0.01, *** *p* < 0.001, and “ns” indicates no significance

The growth medium used for the substrate was composed of a mixture of sandy loam soil, sand, and Turface in a proportion of 1/1/1 (v/v/v) to enhance water drainage and facilitate AMF mycelia recovery at the harvest of the experiment. Sandy loam soil was collected from the organic farm at the L’Institut de Recherche et de Développement en Agroenvironnement (IRDA) in St-Bruno, QC, Canada (45°32’59.6” N, 73°21’08.0” W). This soil was collected from the top 15 cm layer and had a pH value of 6.01, and its characteristics were described in Renaut et al. (2019). After air-drying and sieving at 2 mm, it was combined with sand from Bomix, St-Léonard, QC, Canada, and Turface from Hydro Dionne, Montreal, QC, Canada. Each compartment of the microcosm was filled with approximately 250 mL of substrate.

Leek (*Allium porrum* L., cultivar Monstrous Carentan), a highly mycotrophic host plant, was used in the experiment. The seeds were purchased from McKenzie Seeds (Brandon, MB, Canada). These seeds underwent surface disinfection at room temperature under laminar flow conditions as follows: immersion in 70% ethanol for 30 s, immersion in 15% bleach for 5 min, and thorough rinsing with sterile distilled water. Subsequently, the seeds were soaked in sterile distilled water for 2 h. Pre-germination took place on 1% water agar plates, which were then incubated at 28 °C for one week. Following this, two germinated seeds were placed into each root compartment pot to prevent damaging the radicls.

The germinated seeds were inoculated with 300 µL of a spore suspension in sterile distilled water containing approximately 200 spores of each AMF species separately, as per the experimental design. One month later, one plant per microcosm was retained, and a second inoculation with AMF spores was directly applied to the kept plant roots. To mitigate microalgae contamination, a 1-cm layer of autoclaved Turface was placed atop the microcosms and replaced weekly. The leek plants were cultivated under a 16/8-hour photoperiod and a temperature of 21/18°C (day/night). Additional microcosm units were utilized to monitor plant colonization and the presence of mycelia in RFCs.

Microcosms received weekly watering with half-strength modified Long Ashton nutrient solution with a phosphorus concentration of 22 ppm [35] in the plant compartments to maintain soil moisture at the holding capacity. Irrigation with water occurred two to three times weekly in both compartments (RC and RFC). Two months post-second AMF inoculation, RFC substrate enrichment with a natural soil microbiome was conducted by applying 50 mL of a microbial suspension, as per the experimental design, labeled as treatment B1. For non-inoculated treatments (B0), 50 mL of sterile distilled water was added. This 50 ml volume was determined based water-holding capacity which was measured as 70 mL. Subsequently, the irrigation regime was adjusted for the remainder of the experiment, with a half-strength nutrient solution added weekly to the RFC compartments. Thripex *Neoseiulus cucumeris* (Koppert, Leamington, ON, Canada), a biocontrol agent, was employed for thrip control, and no chemical pesticides were utilized in the experiment.

### Parameters of plant growth, chlorophyll content, and mycorrhizal colonization

Before harvesting the plants, the photosynthetic capacity was determined by measuring the chlorophyll concentration in µg per cm^2^ of the third or fourth fully expanded leaf from the top using an atLEAF chlorophyll meter (FT Green LLC, Washington, USA). Additionally, the stem diameter of the plants was measured using an electronic caliper 5 mm above the sprouting point. The microcosm pots were then carefully separated, and the plants were removed from the substrate. The shoots were detached from the roots and placed in paper bags. The roots were rinsed with sterile distilled water and weighed. A portion of the fine roots was preserved in 8 mL of 50% ethanol for mycorrhizal colonization assessment. The bags containing shoots and the remaining roots were dried at 80 °C for 72 h in a hot-air oven. The remaining roots were weighed to determine the total root dry biomass.

Mycorrhizal colonization was assessed using the ink and vinegar method (Vierheilig et al., 1998). Briefly, the roots were washed with tap water and cut into 1-cm-long segments. These segments were cleared in 10% KOH for 3–4 min at 90 °C, rinsed with distilled water, and then treated with 1% acetic acid for 4 min to remove KOH residues. The cleared roots were stained with a 5% ink–vinegar solution at 90 °C for 3 min and rinsed with distilled water. They were then stored in lactoglycerol solution until microscopic examination using a Zeiss Axio Imager 2 (Zeiss, Montreal, QC, Canada). The stained root segments were placed on microscope slides using fine-point forceps, and the mycorrhizal colonization ratio was determined using the intersection method [35].

### Soil sampling and AMF mycelia collection

To mitigate the influence of surface and root exudates on the AMF hyphae-associated microbial community, the top layer of the RFC substrate (comprising Turface and the upper 1 cm of substrate) was removed along with a 1 cm width from the membrane side. Approximately 10 mL of substrate was then gathered in a 15 mL Falcon tube. We adapted the method used from the International Collection of Vesicular Arbuscular Mycorrhizal Fungi (INVAM) [36], with some modifications, to extract the AMF mycelia from the RFC. The remaining substrate was placed in a sterile 1 L glass jar, covered with cold sterile distilled water, vigorously agitated, and allowed to settle for approximately 15–20 min to aggregate AMF mycelia [36]. Subsequently, the solution was filtered through a sterile sieve with a mesh size of 40 μm. The aggregated mycelia (Figure S2A) were delicately collected from the sieve using fine forceps and transferred into a sterile plate containing approximately 1 mL of cold sterile saline solution (0.9%) made from distilled water and NaCl. This process was repeated four times to maximize the collection of mycelia (Figure S2B). The collected mycelia were then rinsed with cold sterile saline solution (0.9%) to remove any attached debris in a 50 mL Falcon tube (Figure S2C) and then transferred into microcentrifuge tubes, which were gently squeezed to remove excess liquid, and their weights were recorded. Following sampling, all tubes were flash-frozen in liquid nitrogen and kept on dry ice in a cooler box before being stored at − 80 °C. The absence of mycelia in the non-mycorrhizal RFCs (M0) was confirmed by the lack of root colonization by AMF.

### DNA extraction, PCR amplification, and amplicon sequencing of bacterial 16 S rRNA gene

Prior to the DNA extraction process, the soil samples underwent lyophilization for 3 days and were sieved at 1 mm to facilitate the separation of substrate particles [37]. DNA was extracted from 250 mg of homogenized soil from each sample using the DNeasy PowerSoil Pro Kit (Qiagen, Toronto, ON, Canada) following the manufacturer’s instructions. For DNA extraction from the mycelia, the DNeasy Plant Mini Kit (Qiagen, Toronto, ON, Canada) was utilized. Initially, approximately 50 mg of frozen samples were ground in liquid nitrogen in a 1.5 mL tube containing sterile white sand to disrupt the material. Subsequent steps were carried out according to the manufacturer’s guidelines. In the final step of the extraction process, the DNA was eluted in 50 µL of elution buffer for the soil and in 20 µL for the mycelia and then stored at − 20 °C.

Visualization of the extracted DNA was performed using 1% agarose gel electrophoresis stained with GelRed (1/10000) and the GelDoc System (BioRad, Montreal, QC, Canada). Quantification of the DNA was conducted using a Qubit 2.0 Fluorometer and the Qubit double-stranded DNA (dsDNA) HS Assay Kit (ThermoFisher, St-Laurent, QC, Canada).

The V3–V4 region of the bacterial 16S rRNA gene underwent amplification using 341F/806R barcoded primers, as previously described [34, 38]. The primer pair consisted of ***CS1***_341F ***ACACTGACGACATGGTTCTA***CACCTACGGGNGGCWGCAG-3’ and ***CS2***_806R 5′-***TACGGTAGCAGAGACTTGGTCTG***ACTACHVGGGTATCTAATCC-3′) (Farnelid et al., 2019). PCR reactions were conducted in a 25 µL volume mixture comprising 10 µL of Platinum Direct PCR Universal Master Mix (ThermoFisher, St-Laurent, QC, Canada), 0.25 µM of each primer, 4 µL of Platinum GC Enhancer, 8 µL of water, and 1 µL of template DNA. Negative controls containing only water were included in each PCR run. The PCR reactions were carried out using an Eppendorf Mastercycler Pro thermocycler (Eppendorf, Mississauga, ON, Canada) with the following cycling conditions: initial activation at 94 °C for 2 min, followed by 35 cycles of denaturation at 94 °C for 15 s, annealing at 60 °C for 30 s, and extension at 68 °C for 20 s, with a final extension at 68 °C for 1 min and held at 10 °C. Amplification success was confirmed by analyzing the PCR products on a 1% agarose gel. Subsequently, the amplicons were subjected to sequencing on Illumina MiSeq using the paired-end 300 libraries at the Centre d’Expertise et de Services of Genome Quebec (Montreal, QC, Canada).

### Sequence processing and bioinformatic analyses

Bioinformatic analyses were conducted within QIIME2 environment version 2021.4.0 [39]. DADA2 pipeline v1.18.0 [40] was also employed to process the 16 S rRNA gene sequences. The details of filtering and denoising are available in the scripts used by Reneaut et al. [41]. Initially, Cutadapt 3.4 was utilized to remove the primer sequences from the 16 S rRNA gene amplicons, setting parameters such as “minimum-length” at 50, “times = 2”, “overlap = 6”, and “p-error-rate = 0.1”. Subsequently, sequences with less than 220 bp were filtered out using the command “--p-trunc-len”, as the base quality tended to degrade below that threshold in our dataset. Following this, the amplicon sequence variant (ASV) table was generated, and chimeras were removed resulting in a sequence length ranging from 250 to 255 nucleotides. The ASVs were then taxonomically classified using the naive Bayesian classifier method with the SILVA and RDP databases, and the identities of specific ASVs were manually confirmed using BLASTn against the NCBI (nr/nt) database.

### Statistical analyses

Statistical analyses of plant-related parameters, AMF biomass, and mycorrhizal colonization data were conducted using JMP Pro 17 software (SAS Institute Inc., Cary, NC, USA). The normality of the data was assessed using Shapiro–Wilk tests, and variance homogeneity was examined using Levene’s test. An analysis of variance (ANOVA) was then performed on the data for shoot fresh biomass, chlorophyll concentration, and AMF biomass to evaluate the effects of AMF species, microbial addition, and their interactions. However, the data for mycorrhizal colonization were Box–Cox transformed to achieve a normal distribution. AMF inoculation and microbial addition were considered fixed effects, and the random effect was attributed to the block. Tukey’s honestly significant difference (HSD) test was used to compare the mean values that differed at *P* > 0.05.

Microbial analysis was carried out using the vegan v2.6-4 [42], agricolae v1.3-7 [43], and indicspecies v1.7-14 [44] packages in R (version 4.3.2) [45]. The rarecurve function of the vegan package was employed to standardize the dataset by randomly subsampling read data from each sample to the lowest number of reads encountered for a sample. The alpha diversity was assessed by calculating the Shannon and Simpson indices using the vegan package, and the Kruskal–Wallis test was employed to compare alpha diversity between the two biotopes. The significance of AMF identity on alpha diversity was tested using ANOVA, with post hoc tests conducted using the agricolae package. The beta diversity among the samples was computed using the Bray–Curtis distance on bacterial ASVs and visualized with principal coordinate analysis (PCoA) using the vegan package. The community dispersion of bacteria in each biotope was calculated using the “betadisperse” function of the vegan package. The effect of AMF identity and microbial addition on the bacterial community structure was evaluated using PERMANOVA with the Adonis function of the vegan package, employing Hellinger-transformed data and 999 permutations. Indicator species analysis was performed using the indicspecies package to identify the taxa significantly associated with treatments. The term “core bacteriota” was assigned to the set of bacterial taxa present in 90% of hyphae or hyphosphere soil samples associated with the AMF host species [46].

## Results

### Plant response to inoculation with AMF and microbial suspension

The plant fresh and dry biomass, stem diameter, chlorophyll content, mycorrhizal colonization, and AMF biomass are shown in Fig. 2, S2, and S3. Overall, the AMF treatments resulted in a greater increase in plant growth parameters compared to the control (M0), regardless of the amendment to the microbial suspension. Two-way ANOVA revealed that the shoot (*p* < 0.001) and root (*p* < 0.001) fresh weights and stem diameter (*p* < 0.001) were significantly affected by inoculation with AMF spores (Table S1; Figure S2). Similarly, this trend was observed for the shoot (*p* < 0.001) and root (*p* = 0.002) dry weights and chlorophyll concentration (*p* < 0.001) (Table S1; Fig. 2) with an increase of 116.52% and 173.2% in root and shoot dry weights respectively. In contrast, amendment of the microbial suspension only significantly increased the chlorophyll concentration (Table S1; Fig. 2C). Although there was variation in the mean values for mycelial biomass production in the hyphal compartment (RFC), AMF species showed a tendency to influence mycelial biomass production (*p* = 0.067), while the amendment of the microbial suspension was insignificant (*p* = 0.732) (Table S1; Figure S3A). Likewise, the colonization intensity was consistently higher in all treatments, with mean values ranging between 87.41% and 99.56% (Table S1; Figure S3B). For non-inoculated treatments (M0), no mycelia were found in the hyphal compartments, which was attributed to the absence of root colonization in the respective plants. The combined treatments showed a significant effect on chlorophyll content compared to the control (Fig. 2). However, the interactive effect between the AMF spores and microbial inoculation was significant only for the chlorophyll concentration (*p* = 0.05) (Table S1).

**Fig. 3:**
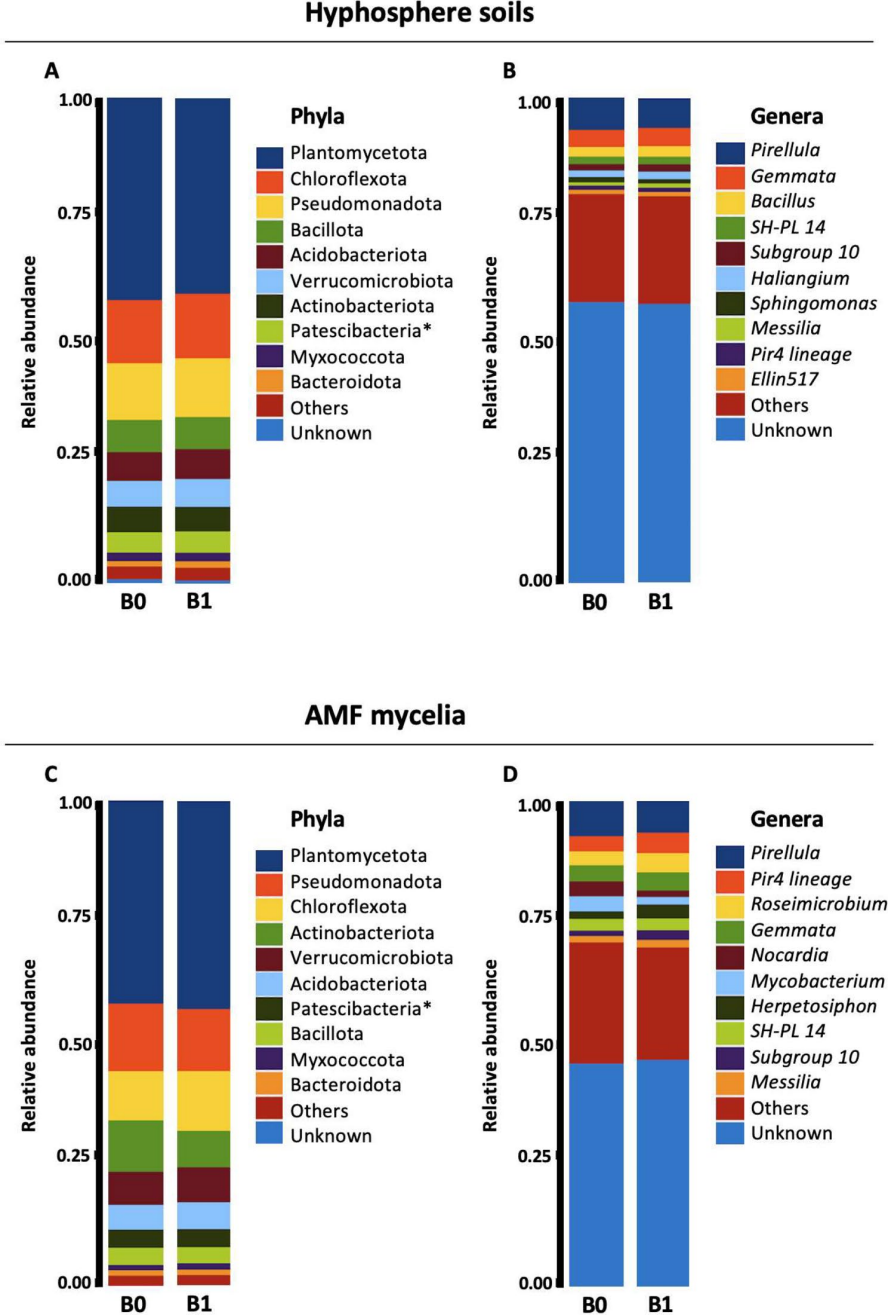
The relative abundances of the 10 most common bacterial taxa in hyphosphere soils (**A** and **B**) and AMF mycelia (**C** and **D**) are depicted in terms of Phylum (**A** and **C**) and Genus (**B** and **D**), with all remaining taxa grouped under “Others”

### Bacterial diversity and composition of AMF mycelia and soil communities

Following Illumina MiSeq sequencing, a total of 10,207,763 bacterial 16 S rRNA gene reads were generated. Through the bioinformatic pipeline, 3,813,362 non-chimeric reads were retrieved from 72 samples after filtering, trimming, denoising, and merging. The reads ranged from 31,424 to 84,914 per sample. These reads were assigned to 8819 bacterial ASVs and further classified into 49 phyla, 119 classes, 297 orders, 386 families, and 965 genera. The rarefaction curves demonstrated that all samples had reached saturation (Figure S4). The bacterial community of AMF mycelia was predominantly comprised of the phylum *Planctomycetota*, representing 42% of the total ASVs, followed by *Pseudomonadota* (13%), *Chloroflexota* (11%), *Actinomycetota* (9%), and *Verrucomicrobiota* (7%). Similarly, *Planctomycetota* constituted the majority of the bacterial soil community, accounting for 41% of the total ASVs, followed by *Chloroflexota* (13%), *Pseudomonadota* (12%), *Bacillota* (7%), and *Acidobacteriota* (6%) (Fig. 3). Among the mycelia, the predominant genera were *Pirellula* (7%), *Gemmata* (4%), Pir4 lineage (4%), and *Roseimicrobium* (2%). Conversely, common genera in the soil were *Pirellula* (6%), *Gemmata* (4%), and *Bacillus* (2%). A substantial proportion of bacteria (47% in the mycelia and 58% in the soil) belonged to unknown genera. Furthermore, 2191 ASVs were exclusively found in the mycelia, 4145 were unique to the soil, and 2051 were shared by both biotopes (Figure S5). Interestingly, at the genus level, the majority of genera were shared between the mycelia and the soil, with 281 genera common to both, 109 found only in the soil, and 44 exclusive to the mycelia (Figure S5).

**Fig. 4:**
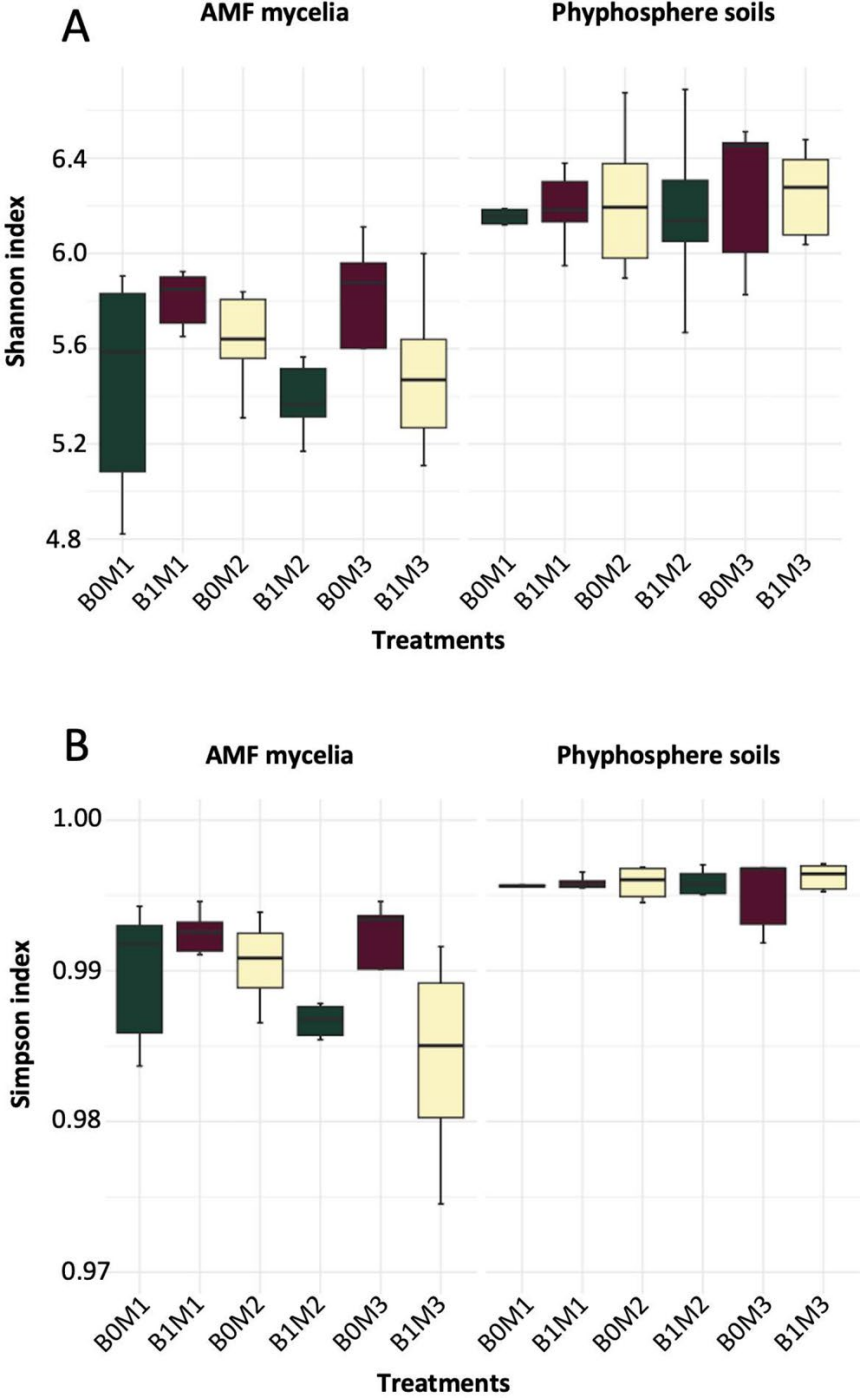
Alpha diversity, assessed using the Shannon (**A**) and Simpson (**B**) indices across various biotopes of AMF mycelia and hyphosphere soils and treatments: microbial amendment (B1), no microbial amendment (B0), *R. irregularis* (M1), *R. clarus* (M2), and *R. cerebriforme* (M3)

### Influence of AMF identity and microbial inoculation on bacterial communities in mycelia and soil

According to the Kruskal–Wallis test, there was a significantly higher alpha diversity in the soil compared to the mycelia (*p* < 0.001). No significant difference in alpha diversity was observed between the B0 and B1 treatments (Table 1; Fig. 4). In both the B0 and B1 treatments, the species of inoculated AMF did not have an impact on the alpha diversity in either the mycelia or the soil. Overall, the alpha diversity remained consistent throughout the experiment when calculated using either the Shannon or Simpson indices.

**Table 1:** Factors significantly influencing the alpha diversity assessed by Shannon and Simpson indices across different biotopes

Sample type	Index	Source of variation	Sum Sq	Mean Sq	NumDF	DenDF	F value	Pr(> F)
AMF	Shannon	AMF	0.262	0.131	2	12.078	1.076	0.372
		MI	0.073	0.073	1	7.368	0.600	0.463
		AMF/MI	0.226	0.113	2	10.934	0.928	0.424
	Simpson	AMF	0.009	0.005	2	12.261	1.036	0.384
		MI	0.004	0.004	1	7.438	0.884	0.377
		AMF/MI	0.009	0.004	2	11.475	0.954	0.414
Hyphosphere, soil	Shannon	AMF	0.572	0.286	2	20.000	0.555	0.583
		MI	0.227	0.227	1	10.000	0.440	0.522
		AMF/MI	0.812	0.406	2	20.000	0.788	0.468
	Simpson	AMF	1.44E-06	7.19E-07	2	20.000	0.323	0.727
		MI	3.25E-08	3.25E-08	1	10.000	0.015	0.906
		AMF/MI	1.84E-06	9.18E-07	2	20.000	0.413	0.667

“AMF” denotes the various inoculated mycorrhizal species, and “MI” represents the B0/B1 treatment of microbial suspension inoculation

**Fig. 5:**
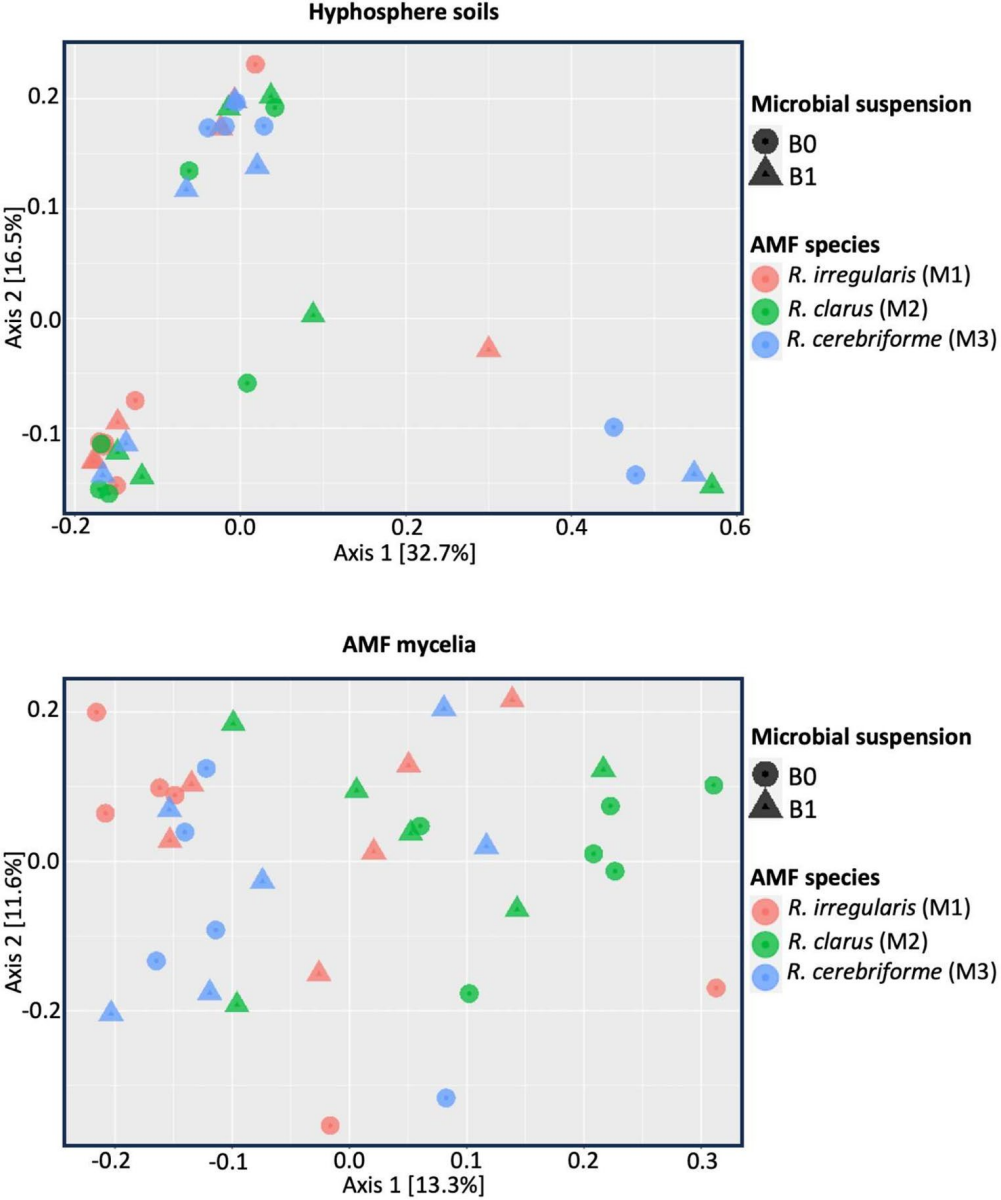
PCoA plots displaying the bacterial communities categorized by their respective biotopes (hyphosphere soils and AMF mycelia), based on the inoculation of AMF species and microbial suspension amendment

We observed significant differences in the bacterial community between the mycelia and the soil (*p* < 0.001). The factors influencing the beta diversity varied between these two environments. In the soil, there were no significant changes in the bacterial community structure between the B0 and B1 treatments or between the AMF species (Table 2; Fig. 5). However, in the mycelia, both the B0/B1 treatments and the inoculated AMF species had a significant effect on the bacterial community structure (*p* = 0.025 and *p* = 0.005, respectively); however, the interaction between microbial treatment and AMF species inoculation was not significant (Table 2; Fig. 5). The “betadisperse” function indicated higher dispersion among the communities in the mycelia (0.42) compared to those in the soil (0.34).

**Table 2:** Factors significantly influencing the beta diversity of bacterial communities including the inoculated mycorrhizal species, represented by “AMF”, and the B0/B1 treatment of bacterial community inoculation, denoted by “MI”. “*” denotes statistical significance, “**” represents high significance, and “^**•**^” indicates a tendency

Sample type	Source of variation	Df	SumOfSq	R2	F	Pr(> F)
AMF mycelia	AMF	2	0.589	0.091	1.687	0.005**
	MI	1	0.230	0.036	1.316	0.025*
	AMF/MI	2	0.413	0.064	1.184	0.055
	Residual	30	5.236	0.810		
	Total	35	6.468	1		
HyphospHere, soil	AMF	2	0.508	0.062	1.095	**0.067** ^**•**^
	MI	1	0.231	0.028	0.997	0.255
	AMF/MI	2	0.455	0.056	0.981	0.29
	Residual	30	6.966	0.854		
	Total	35	8.161	1		

### Indicator species profiles

The analysis of indicator species revealed 126 ASVs across all treatments and sample types (Tables S2–S5). Of these, 77 ASVs (81%) were associated with AMF species treatments (Tables S2 and S4), with 53 ASVs enriched in the mycelia and 24 in the soil. Furthermore, 13 ASVs were linked to the mycelia of *R. irregularis*, 19 to *R. clarus*, and 21 to *R. cerebriforme*. Conversely, six ASVs were enriched in the soil of *R. irregularis* and nine each in the soil of *R. clarus* and *R. cerebriforme*. In contrast, 18 ASVs (19%) were identified as indicator species under microbial suspension amendment, with 10 ASVs enriched in the mycelia and 8 in the soil. Notably, *Herpetosiphon* sp., *Pirellula* sp., and one ASV belonging to the CPla-3 termite group were the most abundant ASVs in the mycelia under microbial amendment (Table S2). However, two ASVs belonging to the phylum *Verrucomicrobiota* (Ellina 516 and *Candidatus Xiphinematobacter*) and one ASV belonging to *Acidobacteriota* were identified in the soil (Table S4). Additionally, *Sulfurifustis* sp., *Pirellula* sp., *Candidatus Omnitrophus*, and *Noviherbaspirillum* sp. were the most dominant ASVs associated with *R. irregularis* mycelia (Table S2). The most abundant ASVs associated with *R. clarus* were *Herpetosiphon* sp., *Fimbriiglobus* sp., two ASVs of *Haliangium* sp., *Rivibacter* sp., and unidentified *Burkholderiales* (Table S2). With the *R. cerebriforme* mycelia, the most predominant ASVs were an unidentified ASV belonging to *Planctomycetota*, another belonging to *Xanthomonadales*, KCM-B-112, which belongs to *Acidithiobacillaceae*, two *Roseimicrobium* sp., and one belonging to the *Burkholderia–Caballeronia–Paraburkholderia* group (Table S2). In the *R. irregularis* soil, an unidentified ASV belonging to the *Gemmataceae* family was the most abundant (Table S5). In addition, one ASV of *Rhodopirellula* was identified in the soil of *R. clarus* (Table S5). However, two ASVs belonging to the Hydrogenispora order and *Blastocatellaceae* family and one to *Nitrosomonadaceae* Ellin6067 stood out in the soil of *R. cerebriforme* (Table S5).

### Common bacterial core in the mycelia of different AMF species and soil

**Fig. 6:**
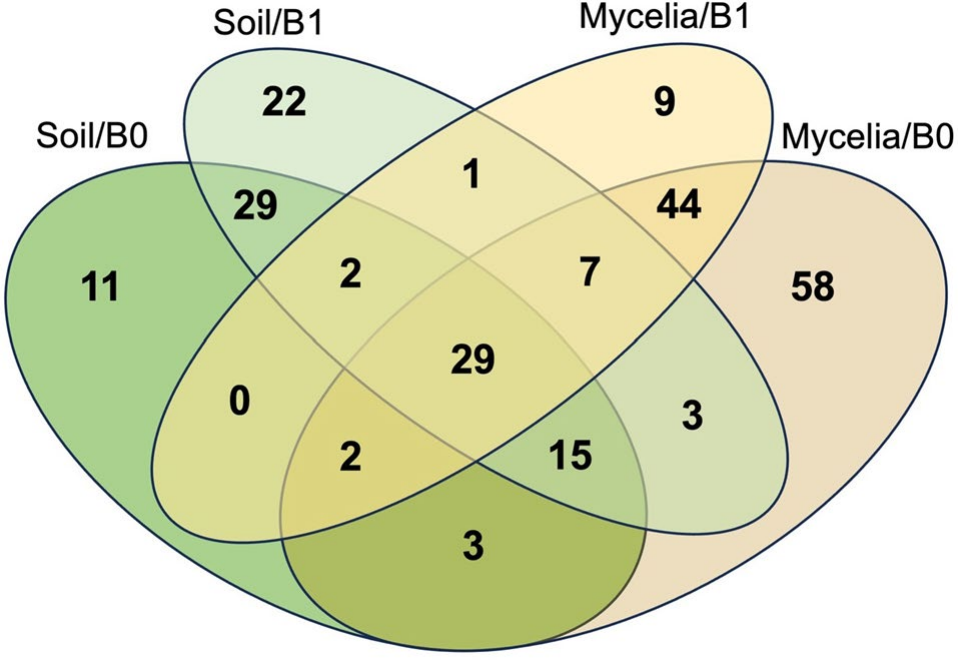
Venn diagram illustrating the core taxa shared between the two biotopes (AMF mycelia and hyphosphere soils) and the two treatments, B1 (microbial inoculation) and B0 (control)

A total of 29 bacterial core taxa were shared among all biotopes and treatments. Interestingly, the majority of these bacterial core taxa (29) were found in the mycelia of both the B0 and B1 treatments, with more core taxa present in the B0 treatment compared to the B1 treatment (Fig. 6). Among the various mycorrhizal species, *R. cerebriforme* (M3) exhibited more core taxa in its mycelia than the other AMF species. However, in the soil, *R. irregularis* (M1) had more core taxa (Figure S6). Most of the core taxa were common to all three AMF species in both the mycelia and the soil, with 78 taxa in the mycelia and 111 in the soil. Interestingly, the bacterial core taxa of *R. cerebriforme* (M3) were greatly increased by B1 treatment, rising from 1 to 138.

## Discussion

The microcosm used in this experiment was designed to investigate the impact of AMF identity on the structure of soil bacterial communities in both enriched and non-enriched microbial environments. Additionally, this provided an opportunity to assess whether the mycelia from different AMF species, which were closely related and cultivated under identical in vitro conditions, would lead to the selection of distinct bacterial communities. This system also facilitated the assessment of the impact of microbial bioaugmentation on plant growth via AMF mycelia.

The predominant bacterial taxa identified in the soil affected by AMF mycelia in the root-free compartment differed from those reported in other studies conducted on the hyphosphere [47, 48] or mycelia [49] of AMF without experimental designs separating them from the roots. Furthermore, the distribution of their abundance differed from that reported in a meta-analysis by Basiru et al. [31] on microbial communities associated with AMF. Interestingly, the dominant bacterial taxa exhibited greater similarity to the bacterial community found in the hyphosphere fungal mats of Basidiomycota species [50], characterized by a prevalence of the phyla *Planctomycetota*, *Chloroflexota*, and *Pseudomonadota*, and at the genus level, an abundance of *Pirelulla*, *Pir4* lineage, *Gemmata*, and *Massilia*. However, the most abundant genera differed between these studies and ours. Consequently, our findings for the soil influenced by the hyphosphere more closely resemble those found in fungal mats compared to those in mycorrhizal fungal communities associated with plant roots. The absence of plant influence due to the microcosm units, separated by two nylon membranes with an opening size of 44 μm, altered the bacterial community. The soil community in our study appeared to consist of taxa belonging to the “mycophilic guild” [50]. Notably, the phylum *Planctomycetota*, which predominated in our study, appeared to be enhanced by the absence of plant roots.

Surprisingly, bioaugmentation using the microbial suspension made from forest soil did not significantly alter the soil bacterial community structure in the root-free compartment.

This result does not corroborate the first hypothesis, which proposed that amending the microbial suspension from natural soil would enhance bacterial diversity and modify the community structure. Overall, the diversity of the soil community appeared to be reduced, as there was also no evidence of AMF species influencing the soil. Another study utilizing a similar compartmented rhizobox system, albeit with a sterilized soil substrate [51], demonstrated very significant differences between the hyphosphere soil communities of different AMF species, contrary to our findings; the overall bacterial community also differed greatly in this study compared to ours. Consequently, AMF seemed to exhibit reduced host specificity in the hyphosphere soil within a soil-based substrate already containing an indigenous microbial community compared to a sterilized one. However, this does not necessarily mean that the AMF had no effect on the overall soil, as demonstrated by the impact of AMF on a forest soil bacterial community [51]. Rather, the hyphosphere soil community appeared to be highly conservative, as evidenced by the fact that microbial inoculation did not alter its composition in our study.

The unexpected variability observed in the mycelial bacterial community when compared to the hyphosphere soil community is another intriguing finding. This result partially supports our second hypothesis. It is possible that AMF mycelia acted as a bacterial highway, facilitating the transportation of microbial communities from the root compartment to the root-free compartment through the nylon membranes. This is supported by de Novais et al. [52], who demonstrated that AMF hyphae facilitated the movement of bacteria between two interconnected plants via the same mycorrhizal network. Another potential mechanism is that AMF mycelia recruited part of their bacterial community from the amendment made by the microbial suspension. These recruited communities did not disperse in the hyphosphere soils but remained attached to the surfaces of the hyphae and spores, which likely explains the shifts observed in the bacterial communities between the mycelia and hyphosphere soils.

Moreover, the taxonomic composition of the bacterial community observed in the mycelia differed significantly from that reported in an AMF inoculum of *R. irregularis* by Agnolucci et al. [53], as well as in another study by the same authors focusing on the spores of other AMF species [53]. Therefore, it can be inferred that substrate composition has an impact on the bacterial communities associated with plant roots [54], which in turn could influences AMF mycelial bacteriome. Certain bacterial taxa, such as *Chloroflexota*, which were abundant in the mycelia in our study, were not present in the spores in these two studies, suggesting a context-specific association with mycelia development in the soil. Despite the substantial beta diversity difference observed between the hyphosphere soils and mycelia, a significant number of ASVs, as well as the majority of bacterial genera, were shared between the two biotopes, indicating a certain degree of connectivity between them. Consequently, our findings suggest a higher level of host selectivity within the mycelia compared to the hyphosphere soil, and/or a lack of consistency in the mycelia bacterial community, which may result in some stochastic effects, consistent with the increased dispersion observed in mycelia communities.

The bacterial community profiles associated with different AMF species varied. In particular, the species *R. cerebriforme* exhibited a strong correlation with three bacterial genera, including *Glomeribacter*, known for forming symbiotic associations with AMF species [55]. Additionally, *R. clarus* and *R. irregularis* each showed a correlation with one bacterial genus. These data support the third hypothesis, suggesting that while all AMF species establish some level of association with certain bacterial taxa, the primary selection process predominantly occurs within the mycelia and is less pronounced in hyphosphere soils. The observed variation in the bacterial community selection by AMF aligns with previous findings, indicating that some AMF taxa possess a higher enrichment of secreted protein genes compared to other species [10], which may lead to a more selective association with the bacterial community. Although the three AMF species belong to the same genus *Rhizophagus*, with *R. irregularis* and *R. clarus* being closely related compared to *R. cerebriforme*, we observed variations in the bacterial communities associated with their mycelia, suggesting the organismal phylogeny influence of bacterial communities associated with AMF mycelia. Notably, the mycelial bacterial community of *R. cerebriforme* contained 60 bacterial core taxa exclusive to it, while *R. clarus* and *R. irregularis* had 25 and 9 exclusive taxa, respectively. This supports the hypothesis that *R. cerebriforme* exhibits a highly selective association with its bacteriome, whereas *R. irregularis* demonstrates a comparatively weaker selection. Further research employing a variety of AMF species from different genera and families, coupled with both taxonomic and functional analyses, is required to elucidate the impacts of organismal phylogeny and life history strategies on the recruitment of bacterial communities by AMF.

## Conclusions

Our study employed a meticulously designed microcosm to explore how the identity of AMF influences the soil bacterial community structure in both enriched and non-enriched microbial environments. We also delved into whether mycelia from closely related AMF species cultivated under similar conditions would selectively shape distinct bacterial communities. Additionally, we evaluated the impact of AMF species, microbial bioaugmentation and their interactions on plant growth through AMF mycelia.

Our findings unveiled notable disparities in the predominant bacterial taxa between soil influenced by AMF mycelia and those reported in previous studies on AMF hyphospheres or mycelia without experimental designs separating them from the roots. Intriguingly, our study’s bacterial communities closely resembled those found in hyphosphere fungal mats compared to those in mycorrhizal fungal communities associated with plant roots, indicating a significant alteration due to the absence of plant roots in our experimental setup.

Unexpectedly, microbial bioaugmentation using a forest soil-derived microbial suspension did not significantly alter the soil bacterial community structure in the RFC. Furthermore, we observed a reduction in overall soil community diversity, suggesting no apparent influence of AMF species on the soil.

The unexpected variability observed in the mycelial bacterial community compared to the hyphosphere soil community suggests that AMF mycelia may serve as a conduit for microbial transportation between compartments. Additionally, our study highlighted the impact of substrate composition on the bacterial communities associated with AMF mycelia. Furthermore, we observed a higher level of host selectivity within mycelia compared to hyphosphere soil, suggesting a more tailored interaction between AMF and the bacterial communities within mycelia.

The distinct bacterial community profiles associated with different AMF species further support the hypothesis that selective association processes occur primarily within mycelia. This selective process appears to be influenced by organismal phylogeny, as evidenced by the variations observed in the bacterial communities associated with different AMF species.

Furthermore, the results of this study highlight the important role of AMF species and their associated bacterial communities in enhancing plant growth. The three AMF species not only increased shoot and root biomass but also elevated chlorophyll concentration in the shoots, demonstrating their potential to boost plant productivity. These findings have significant implications for agricultural and ecological management strategies.

Our study emphasizes the critical role of considering AMF identity and microbial bioaugmentation in shaping soil bacterial communities. Further research incorporating a diverse range of AMF species from various genera and families, coupled with comprehensive taxonomic and functional analyses, is warranted to elucidate the intricate interactions between AMF and bacterial communities. Ultimately, understanding these interactions is essential for harnessing the potential of AMF inoculants to enhance plant growth and ecosystem services.

## Electronic supplementary material

Below is the link to the electronic supplementary material.

Supplementary Material 1

Supplementary Material 2

## Data Availability

The raw reads corresponding to the hyphosphere soil samples and the AMF mycelia samples were submitted to NCBI (https://www.ncbi.nlm.nih.gov/), with accession numbers provided under the BioProject PRJNA1105031.
