# Supplementary material for: The composition of the arbuscular mycorrhizal fungal bacteriome is species dependent
Source: Environ Microbiome. 2024 Oct 16;19:77. doi: 10.1186/s40793-024-00623-z (PMC11484372; doi:10.1186/s40793-024-00623-z)
Supplement: Supplementary file 1 — Supplementary Material 1 [file 40793_2024_623_MOESM1_ESM.docx]

**Supplementary Information**

**Table S1:** Effects of AMF identity and microbial inoculation on the root fresh weight (RFW), shoot fresh weight (SFW), root dry weight (RDW), shoot dry weight (SDW), stem diameter (SD), chlorophyll concentration (CHL) and mycorrhizal colonization (MC) of leek plants and AMF biomass production of the three AMF species.

|  | DF | RFW | |  | SFW | | |  | | RDW | |  | SDW | |  | StemD | |  | CHL | |  | AMF biomass | |  | MC | |
| --- | --- | --- | --- | --- | --- | --- | --- | --- | --- | --- | --- | --- | --- | --- | --- | --- | --- | --- | --- | --- | --- | --- | --- | --- | --- | --- |
|  |  | *F* | *p* |  | *F* | *p* |  | | *F* | | *p* |  | *F* | *p* |  | *F* | *p* |  | *F* | *p* |  | *F* | *p* |  | *F* | *p* |
| AMF species | 3 | 21.552 | **<0.001** |  | 50.086 | **<0.001** |  | | 8.009 | | **0.002** |  | 16.153 | **<0.001** |  | 14.712 | **<0.001** |  | 41.957 | **<0.001** |  | 3.597 | 0.067 |  | 1.371 | 0.298 |
| Microbial inoculation | 1 | 0.0001 | 0.992 |  | 0.456 | 0.530 |  | | 0.080 | | 0.788 |  | 0.460 | 0.528 |  | 0.526 | 0.501 |  | 44.421 | **0.001** |  | 0.131 | 0.732 |  | 1.078 | 0.347 |
| AMF x MI | 3 | 0.858 | 0.484 |  | 0.541 | 0.662 |  | | 0.218 | | 0.883 |  | 0.216 | 0.884 |  | 0.284 | 0.836 |  | 3.262 | **0.051** |  | 0.428 | 0.663 |  | 0.351 | 0.713 |
|  |  |  |  |  |  |  |  | |  | |  |  |  |  |  |  |  |  |  |  |  |  |  |  |  |  |

**Tables S2-S5** are included in a separate Excel file.

Figure S1


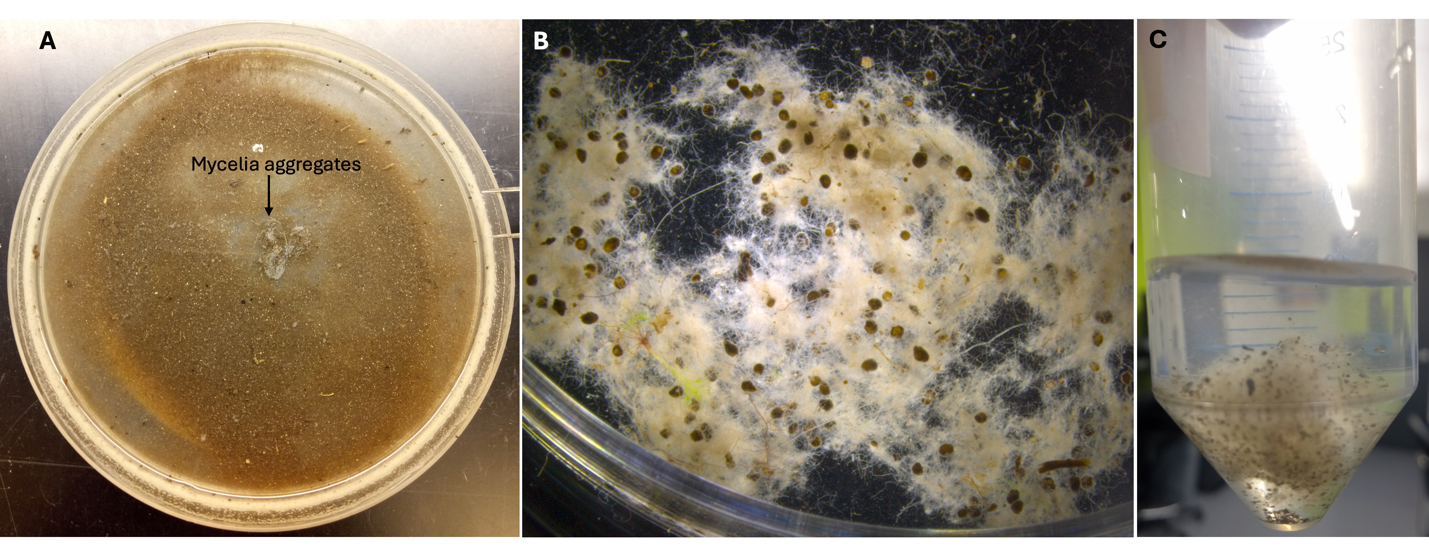


**Figure S1:** AMF mycelium harvested from the root-free compartment. Panel A shows an aggregate of extraradical mycelium after wet-sieving. Panels B and C display AMF extraradical mycelium, composed of spores and hyphae, after washing and cleaning.

Fig. S2


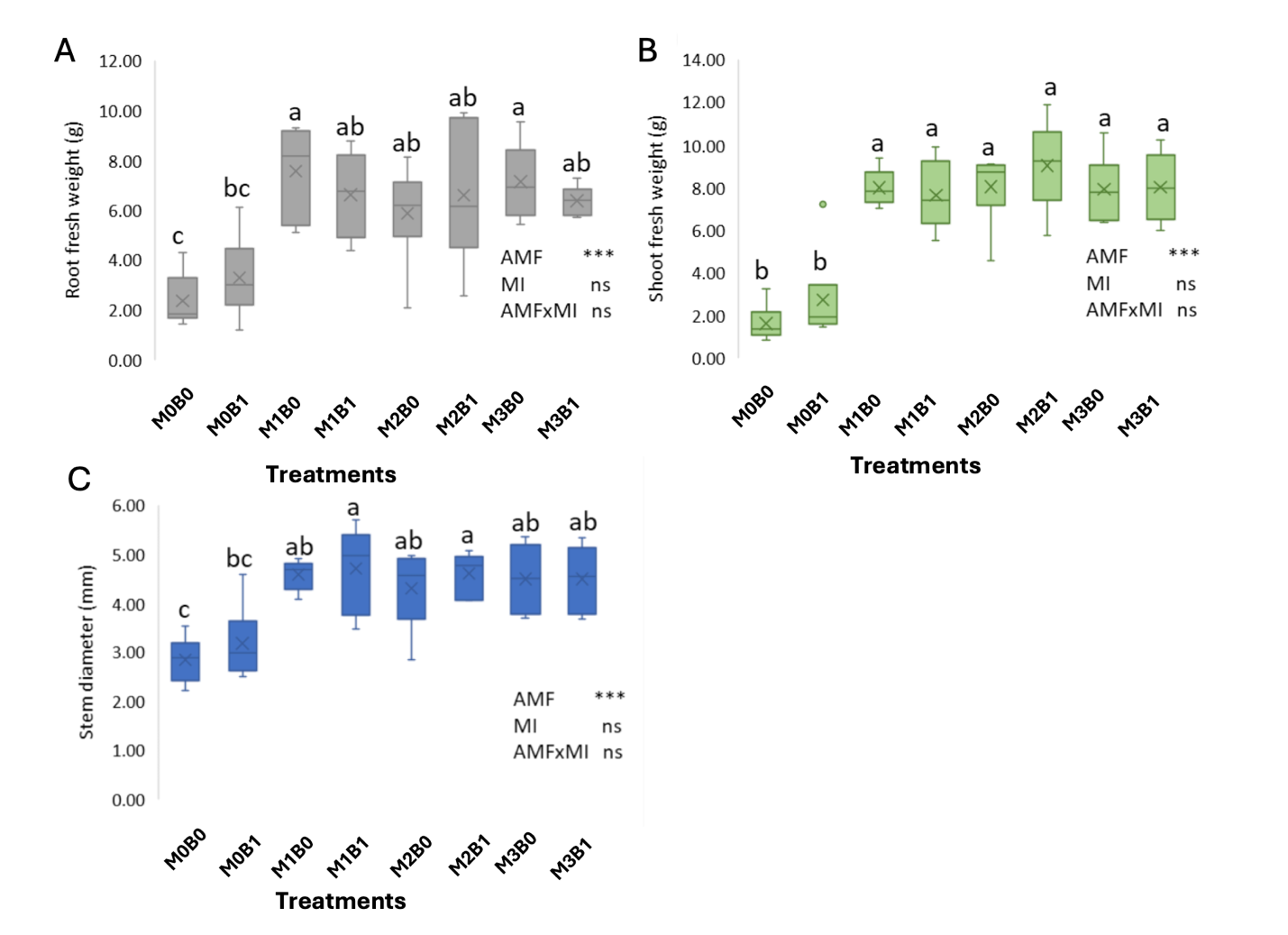


**Figure S2:** The influence of AMF identity and microbial inoculation on root fresh (A), shoot fresh weight (B) and stem diameter (c) was examined across the three AMF species. Significance levels are denoted as follows: * if p < 0.05, ** if p < 0.01, *** if p < 0.001, and "ns" indicates no significance.

Fig. S3


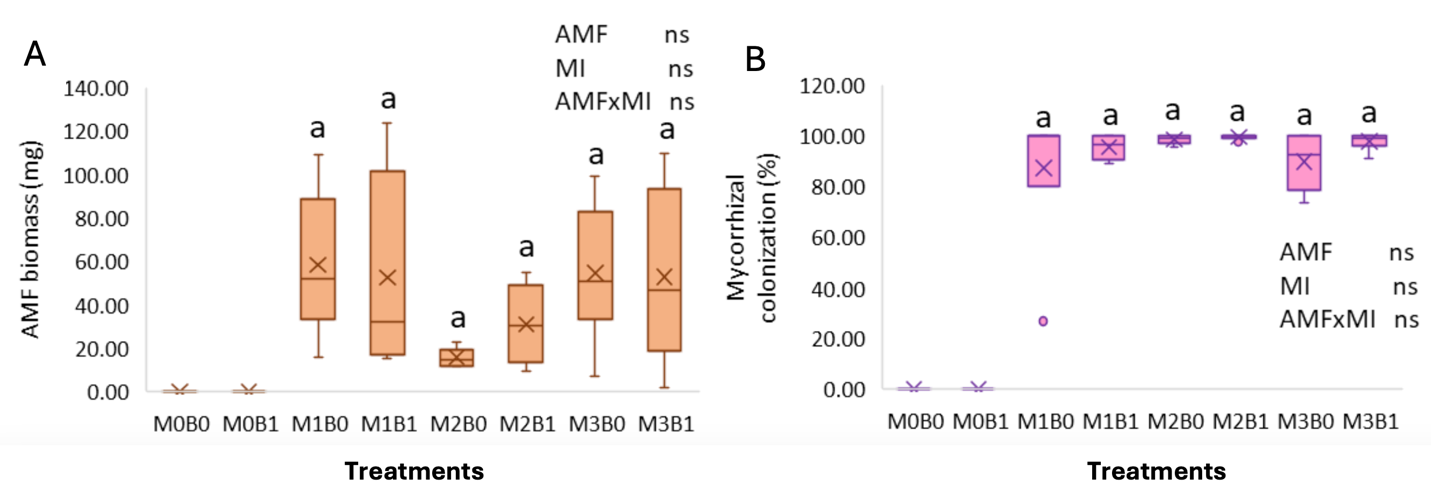


**Figure S3:** The influence of AMF identity and microbial inoculation on both AMF mycelia production (A) and root mycorrhizal colonization (B) was examined across the three AMF species. "ns" denotes non-significance.

Fig. S4


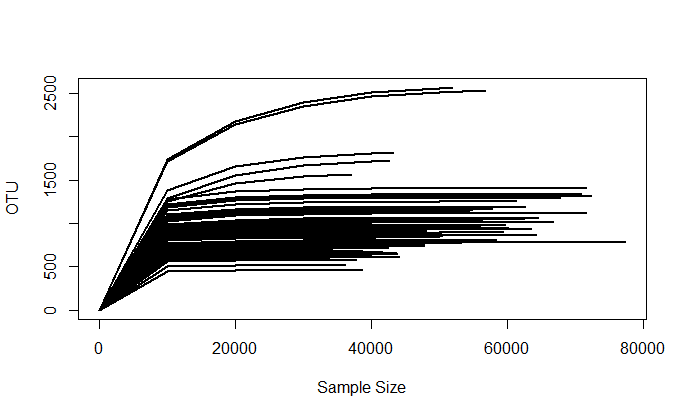


**Figure S4:** Rarefaction analysis was conducted for each sample, revealing that all curves reached saturation.

Fig. S5


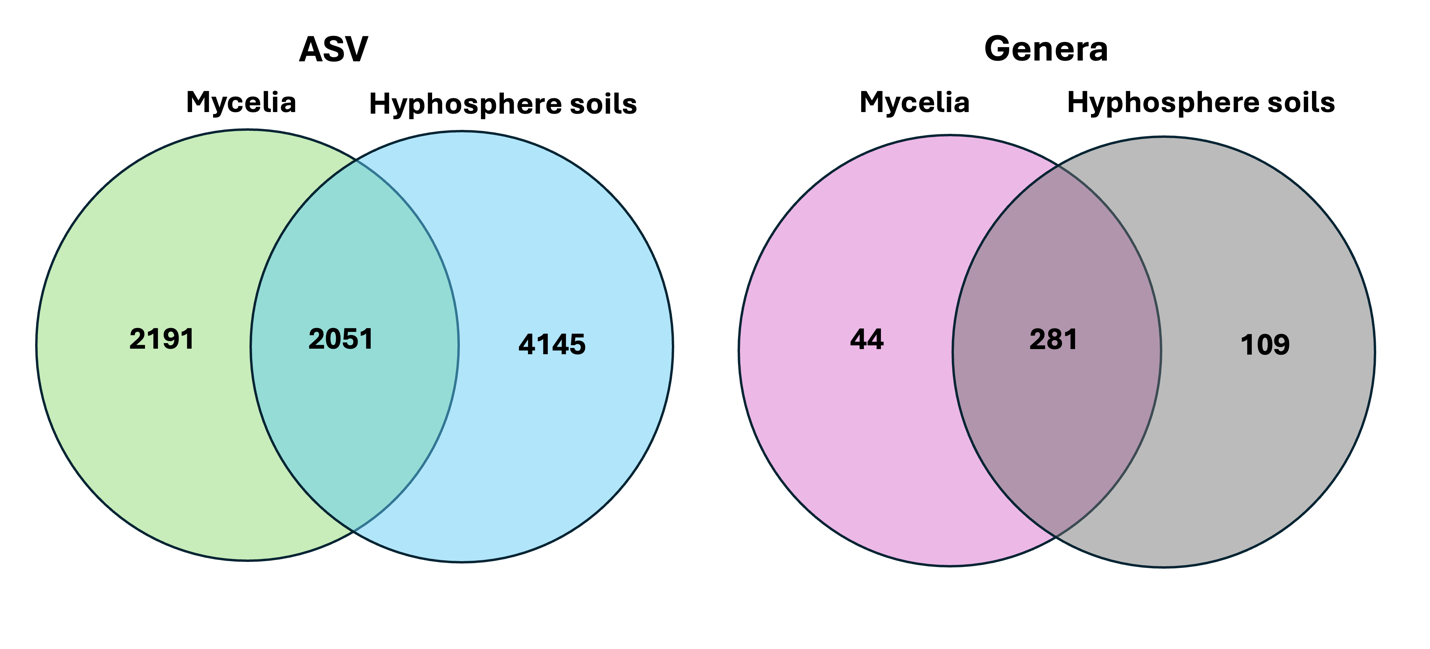


**Figure S5**: Venn diagrams illustrating the taxa shared between hypha and hyphosphere at both the ASV and genus levels.

Fig. S6


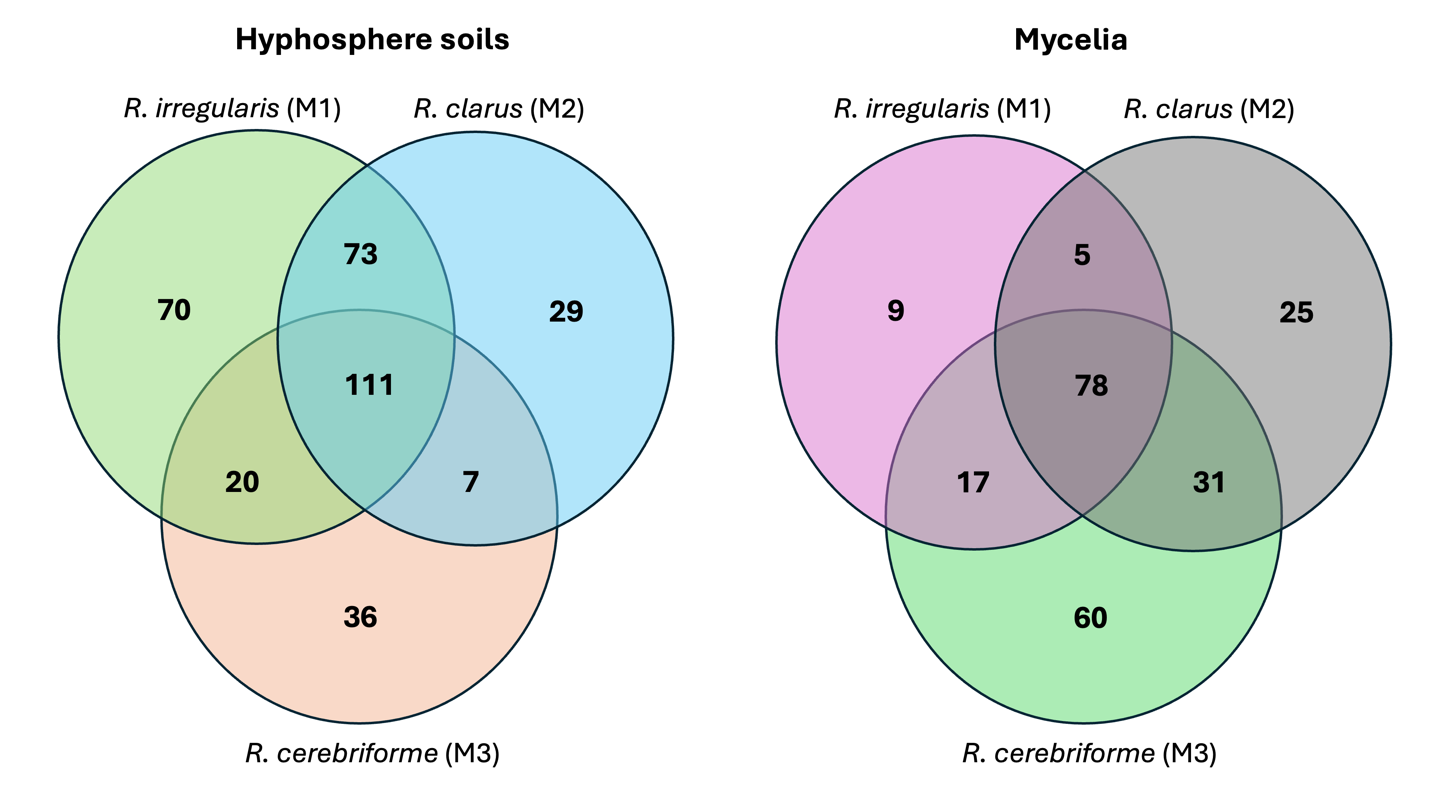


**Figure S6:** A Venn diagram depicting the core taxa shared among the three mycorrhizal species and the two biotopes (mycelia and hyphosphere soils) at the ASV level.
